# The impact of anticipated stigma on psychological and physical health problems in the unemployed group

**DOI:** 10.3389/fpsyg.2015.01263

**Published:** 2015-08-25

**Authors:** Aisling T. O’Donnell, Fiona Corrigan, Stephen Gallagher

**Affiliations:** Department of Psychology and Centre for Social Issues Research, University of LimerickLimerick, Ireland

**Keywords:** anticipated stigma, stigmatization, psychological distress, depression, anxiety, physical health symptoms, unemployment, unemployed identity

## Abstract

Previous research has demonstrated that the unemployed suffer increased psychological and physical health problems compared to their employed counterparts. Further, unemployment leads to an unwanted new social identity that is stigmatizing, and stigma is known to be a stressor causing psychological and physical health problems. However, it is not yet known whether being stigmatized as an unemployed group member is associated with psychological and physical health in this group. The current study tested the impact of anticipated stigma (AS) on psychological distress (PD) and physical health problems, operationalized as somatic symptoms (SSs), in a volunteer sample of unemployed people. Results revealed that AS had a direct effect on both PD and SSs, such that greater AS significantly predicted higher levels of both. Moreover, the direct effect on SSs became non-significant when PD was taken into account. Thus, to the extent that unemployed participants anticipated experiencing greater stigma, they also reported increased PD, and this PD predicted increased SSs. Our findings complement and extend the existing literature on the relationships between stigmatized identities, PD and physical health problems, particularly in relation to the unemployed group. This group is important to consider both theoretically, given the unwanted and transient nature of the identity compared to other stigmatized identities, but also practically, as the findings indicate a need to orient to the perceived valence of the unemployed identity and its effects on psychological and physical health.

## Introduction

Previous research has demonstrated that the unemployed suffer increased health problems compared to their employed counterparts. This is important as the aftereffects of the global financial crisis mean that unemployment rates are still high at the time of writing. In Ireland, the context of the current paper, the unemployment rate was 9.9% for the first quarter of 2015 (CSO, 2015), representing a large increase on 10 years previously (only 4.2%; CSO, 2005) and higher than the harmonized average for the OECD area for mid-2014 of 7.3% (OECD, 2014). Such high levels of unemployment can have far-reaching effects on health: there is evidence that unemployment is associated with both increased psychological health problems (Paul and Moser, 2009; Eriksson et al., 2010; Blau et al., 2013; Breslin and Breslin, 2013) and increased physical health problems, including self-rated physical health, illness and symptoms (McKee-Ryan et al., 2005) and psychosomatic disorders such as headache, stomach ache and sleeping disorders (Linn et al., 1985; Rantakeisu et al., 1997). Current known or hypothesized reasons for this include the impact of financial strain (Breslin and Breslin, 2013), stress associated with income loss (Eriksson et al., 2010), but also latent deprivation of employment-related functions (time structure, social contact, collective purpose, status, and activity; Jahoda, 1982; see also Creed and McIntyre, 2001). There is also a more general proposition that unemployment is in itself a stressful event (Linn et al., 1985; McKee-Ryan et al., 2005; Eriksson et al., 2010). Following on from this, and as noted in the literature (Eriksson et al., 2010; Quinn and Earnshaw, 2011), one missing piece of the puzzle in explaining health disparities between advantaged and disadvantaged groups – such as the unemployed – may be the effect of stigma. Stigma is the social devaluation of a person or group on the basis of some characteristic (Goffman, 1963) – in this case, unemployment-related stigma. To expand on this, stigmatization involves labeling someone as different, associating them with some negative stereotype on this basis, and discriminating against them (Link and Phelan, 2006). To date, the research on stigma and health among the unemployed is sparse, and given the negative impact of stigma for other stressed groups (Kaur and Van Brakel, 2002; Nyblade et al., 2003; Jacoby et al., 2004), it is worthy of investigation.

While all individuals are said to have a varied self-concept that is partly made up of many different social identities, some chosen (e.g., scientist) and some ascribed at birth (e.g., woman; Tajfel and Turner, 1979), other identities, such as unemployment, are thrust upon them. It is known that unemployment leads to an unwanted new social identity that is stigmatizing (Cullen and Hodgetts, 2001; Letkemann, 2002). Further, studies at both the population and individual level show that employers discriminate by declining to employ those who are already unemployed. In some of these studies it is assumed that there is a ‘stigma effect’; that is, that the unwillingness to hire the unemployed is driven by inferences that their unemployment is due to personal failings (e.g., Oberholzer-Gee, 2008; Biewen and Steffes, 2010; Contini and Richiardi, 2012). However, in other studies, people’s stigmatized views of the unemployed have been directly measured (e.g., Furåker and Blomsterberg, 2003) and indeed greater stigmatization of the unemployed has been shown to predict reduced willingness to employ them (e.g., Ho et al., 2011; see also Karren and Sherman, 2012, for a review). Consistent with this evidence, there has also been research focusing on the experience of unemployment, which demonstrates that the unemployed perceive it as stigmatizing (e.g., Rantakeisu et al., 1997; Kulik, 2000; Cullen and Hodgetts, 2001; Letkemann, 2002; Lee et al., 2005). Despite these studies showing the unemployed experience stigma, to our knowledge, the association between stigma and health in this group has yet to be examined.

Stigma is widely understood to be a stressor causing psychological and physical health problems. We know from previous research that stress in general impacts well-being. For example, it has been shown that stress can lead to psychological distress (PD; e.g., Schulz et al., 1995; Phillips et al., 2015) and that collectively, stress and PD can impact physical health (e.g., Cohen and Williamson, 1991; Hoge et al., 2007; Springer et al., 2007; Springer, 2009; Gallagher and Whiteley, 2013). Particularly relevant to the current study is the large body of evidence related to stigmatized identities other than unemployment, demonstrating that these stigmatized identities impact negatively on health and well-being in terms of: depression and/or anxiety (Markowitz, 1998; Meyer, 2003; Link and Phelan, 2006; Cluver et al., 2008; Quinn and Chaudoir, 2009; Quinn et al., 2014), post-traumatic stress (Katz and Nevid, 2005; Cluver et al., 2008), reduced quality of life (Earnshaw and Quinn, 2011; Earnshaw et al., 2011), reduced self-esteem (Chaudoir and Quinn, 2010), negative affect (Hatzenbuehler et al., 2009) and poor physical health, such as increased illness symptoms (e.g., chest pain, nausea, coughing; Quinn and Chaudoir, 2009), and even chronic illness comorbidity and low CD4 count in individuals with HIV/AIDS (Earnshaw et al., 2013). Importantly, many of these studies refer to stigmatized identities that are concealable, such as HIV/AIDS, mental illness and certain chronic diseases. Unemployment is also a concealable stigmatized identity, but it has not yet been clearly demonstrated how stigmatization associated with this particular identity is associated with increased PD and physical health problems.

At this point it should be noted that in the case of some stigmatized identities, it is clear that physical health problems precede stigmatization and PD because the stigma relates to a physical health problem (e.g., in the case of HIV/AIDS) which then leads to PD. In the case of unemployment, while there has been some suggestion that people experiencing ill health disproportionately self-select into unemployment, there is also evidence that there is a detrimental effect of unemployment on physical health that is not caused by self-selection (Korpi, 2001; Wanberg, 2012). Moreover, it is possible to investigate whether unemployment precedes psychological and physical ill health by controlling for illnesses experienced prior to the onset of unemployment.

It has not yet been demonstrated whether the belief that one is being stigmatized as an unemployed group member might drive the negative impact of unemployment on psychological and physical health. The current study will address this gap in the literature. Given that the anticipation of stigma has been identified as particularly relevant in studies of other concealable stigmatized identities (e.g., Earnshaw and Quinn, 2011; Earnshaw et al., 2011, 2013), and given that it can be even more disruptive to people’s lives than experienced discrimination (Gilbert and Walker, 2010), the current study will focus on the impact of anticipated stigma (AS) associated with unemployment. We measure PD as a composite of depression and anxiety, both outcomes that have been identified as important and relevant in the literatures on unemployment (Linn et al., 1985; McKee-Ryan et al., 2005; Paul and Moser, 2009; Wanberg, 2012), stress (Schulz et al., 1995; Springer et al., 2007), and stigma (Markowitz, 1998; Cluver et al., 2008; Quinn and Chaudoir, 2009; Quinn et al., 2014). Finally we operationalize physical health via self-report of somatic symptoms (SSs), again a commonly used measure of physical health complaints (e.g., Hoge et al., 2007; Springer et al., 2007). We then test whether higher levels of AS are associated with increased PD and SSs (i.e., physical health problems) and also whether any effect of AS on SSs is carried by the effect of AS on PD.

## Materials and Methods

### Participants

Forty-eight people based in a small city in Ireland and currently experiencing unemployment took part in the study (15 male, 33 female). Their ages ranged from 18 to 65 years (*M* = 33.49, SD = 13.14; one participant did not indicate their age). The majority of participants (87.5%) reported that they had not been diagnosed with any mental or physical illness before becoming unemployed. Participants had been unemployed for a minimum of 1 month and a maximum of 120 months (10 years). The mean length of time unemployed was 20 months (1 year and 8 months; SD = 28.99). In terms of education level, participants ranged from those who had attended some secondary school but not completed it, to those with postgraduate degrees. The most common level of education among the sample was holding an undergraduate degree. Most participants were either single (45.8%) or married/cohabiting (43.8%), with a smaller proportion reporting being either separated/divorced (6.3%), widowed (2.1%), or declining to indicate marital status (2.1%). Household income for the participants ranged from less than €20,000 to somewhere between €60,001 and 80,000; however, the modal income bracket was the lowest one (€0–20,000).

### Procedure

Ethical approval was granted by our Faculty’s Research Ethics Committee, and indeed the research was conducted in line with the ethical principles of the Declaration of Helsinki and the American Psychological Association (APA, 2010). Participants were recruited to take part in the study either online, via advertisements circulated on Twitter and Facebook, or in person at Social Welfare offices, and through groups and organizations geared toward helping the unemployed. It was possible to complete the survey either online, using Questback software, or using a pen-and-paper version of the questionnaire, depending on the participant’s preference. Online advertisements included a link to the online survey, but those recruited online could still opt to receive a paper version of the survey if preferred, and likewise those recruited in person could also choose the online version. By necessity, those recruited in person had some contact with the research team, although they completed the surveys themselves, while those recruited online had no contact. All participants were informed that the study was investigating the health outcomes of unemployment. Participants indicated their informed consent either by signing or by ticking a box, depending on their mode of participation. Participants did not receive compensation for completing the survey.

### Measures

Participants first responded to demographic items and then completed the following measures, all of which are reliable and valid as outlined below. As indicated in the Introduction, AS was measured as a predictor variable, PD as a mediator variable, and SSs as an outcome variable.

#### Anticipated Stigma

An adapted version of the Day-to-Day Discrimination scale (Kessler et al., 1999) was used to measure AS. A similarly adapted measure has previously been used to assess AS amongst a sample possessing various concealable stigmatized identities (Quinn and Chaudoir, 2009). The original scale lists nine examples of discrimination, and participants must respond how often these have occurred to them in the past. In the present study, the same nine items were used, but the instruction was adapted to ask participants to indicate how likely or unlikely they think each one would be to occur if people knew about their unemployment status. In this way, the measure was used to capture the extent to which participants anticipated being socially stigmatized if they were to reveal their unemployed identity. Two example items were “People acting as if you are inferior” and “Being treated with less respect than others.” Participants indicated their responses on a scale ranging from 1 (Not at all likely) to 5 (Very likely). Total scores can range from 9 to 45 with higher scores indicating greater AS. This scale has shown good internal reliability and construct validity in previous studies (Paradies, 2006), and in the current study also demonstrated high internal reliability (α = 0.90).

#### Psychological Distress

Psychological distress was measured by assessing participants’ levels of depression and anxiety using the Hospital Anxiety and Depression Scale (HADS; Zigmond and Snaith, 1983). This measure has been demonstrated as effective in assessing the severity of anxiety and depression symptoms in the general population as well as psychiatric and primary care patients (Bjelland et al., 2002), and has also been utilized in previous research with unemployed samples (Berth et al., 2003; Limm et al., 2012). In the present study, internal reliability was very good (α = 0.91). Responses to this 14-item scale were indicated on four point scales ranging from 0 to 3, although anchors varied depending on the item. Seven items were reverse scored so that higher scores denoted more PD (scores can range from 0 to 42).

#### Somatic Symptoms

Participants’ experience of SSs was measured using the 14-item Physical Health Questionnaire (PHQ; Schat et al., 2005). Participants indicated how often they experienced various SSs, including headaches, constipation/diarrhea and colds, over the last year using a scale ranging from 1 (Not at all) to 7 (All of the time). One item was reverse scored, such that higher scores denoted more impaired physical well-being. In the present study, all items were summed into one total score, which has been done in previous research (e.g., Schat and Kelloway, 2003; Arnold and Dupré, 2012). Total scores can range from 14 to 98. As noted by Dupré and colleagues, self-perceived health status has been shown to accurately predict actual health outcomes in prior research (Dupré and Day, 2007; Arnold and Dupré, 2012). This particular scale has been used to measure somatic health in other distressed samples (Cantwell et al., 2014) and has also been employed in previous research with an unemployed sample (Carnicella, 2013). In the present study it demonstrated high internal reliability (α = 0.88).

### Data Analysis

The impact of AS on both PD and SSs was tested using simple linear regression analyses. Bootstrapping methods (Hayes, 2009) were then used to determine whether the effect of AS on SSs might be partly accounted for by the effect of AS on PD. We chose bootstrapping because it allows for the analysis of data that are not normally distributed, and because it works by taking repeated samples from the dataset, thousands of times, and estimating the indirect effect with each of these resampled datasets. This is particularly useful for datasets that are relatively small, such as this one. We used IBM SPSS 20 to analyze the data, and to carry out the mediation analysis we used the custom dialog PROCESS for SPSS by Hayes (2013).

## Results

### Descriptive Statistics

**Table 1** presents the means, standard deviations and correlations between AS, PD, and SSs. It also contains data on potential confounds, i.e., the socio-demographic variables discussed earlier. Mean levels of AS were moderate, being slightly below the mid-point of the scale, which is comparable to studies of other stigmatized identities (e.g., Earnshaw et al., 2013; Quinn et al., 2014). Mean levels of PD would be classified as mild (Zigmond and Snaith, 1983). Classifications are not available for the PHQ, but the mean score is just under the mid-point and is comparable with levels observed in other recent studies (e.g., Cantwell et al., 2014) so can be considered moderate.

**Table 1:** Intercorrelations, means, and standard deviations of the measures of this study.

	1	2	3	4	5	6	7	8	9	*N*	*M* (SD)
1. Anticipated stigma	–	0.63^∗∗^	0.42^∗^	0.13	0.13	-0.04	-0.09	-0.11	-0.14	48	20.81 (7.75)
2. Psychological distress		–	0.66^∗∗^	-0.14	0.33^†^	-0.09	-0.05	-0.19	-0.11	48	14.81 (8.66)
3. Somatic symptoms			–	0.16	0.33^†^	-0.02	-0.11	-0.12	0.00	48	45.69 (13.93)
4. Prior illness				–	0.07	-0.11	-0.02	0.16	0.15	48	–
5. Duration unemployed (months)					–	-0.33^†^	0.49^∗^	-0.16	0.17	34	20.00 (28.99)
6. Highest educational level						–	-0.22	0.19	0.20	48	–
7. Marital status							–	0.06	-0.09	47	–
8. Household income								–	0.25	48	–
9. Sex									–	48	–

*^∗∗^Correlation is significant at the 0.001 level; ^∗^correlation is significant at the 0.01 level; ^†^correlation is marginal (p ≤ 0.06)*.

As can be seen below, AS, PD, and SSs were significantly correlated with one another. These associations were positive, such that unemployed people who reported higher AS reported both greater PD and more SSs; similarly, those who reported higher PD also reported more SSs. There were no significant correlations between these variables and any of the potentially confounding variables, but the duration of unemployment in months was marginally associated with PD and SSs.

### Testing the Effect of Anticipated Stigma on Somatic Symptoms as Mediated by Psychological Distress

The aim of this analysis was to test whether the direct effect of AS on SSs would be mediated by PD. We tested for mediation by regressing the predictor variable, AS, on the outcome variable, SSs, while also including the proposed mediator, PD. We first conducted these analyses including the six potential confounds identified above as covariates, specifically because one of them was found to be marginally associated with two of the key measures. None of the covariates was significant in the model, and the results were the same as when we conducted the analyses without the covariates. As such, here we report the results without controlling for these variables, as it allows us to include the Kappa squared (κ^2^) effect size (Preacher and Kelley, 2011), which cannot be calculated for models that include covariates.

**FIGURE 1:**
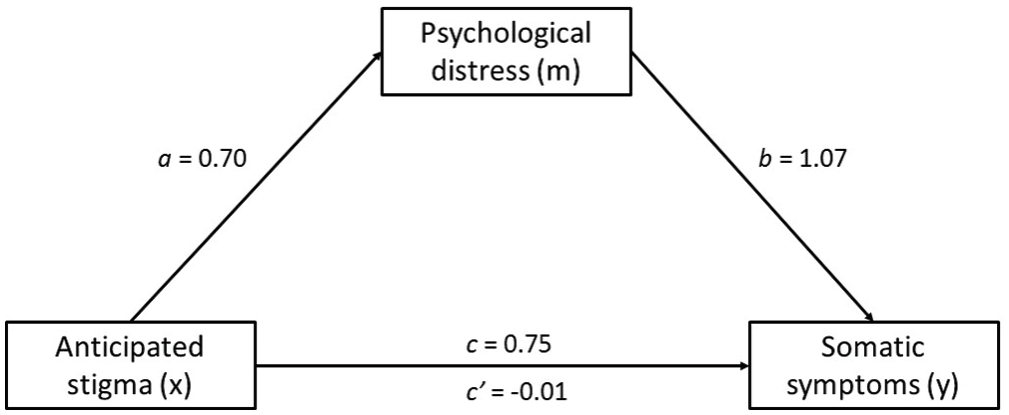
**Mediation of the effect of anticipated stigma on somatic symptoms by psychological distress**.

The mediation analysis was conducted using 5000 bootstrap samples and confirmed that there was a significant direct effect of AS on SSs, but that as predicted, this was rendered non-significant when the effect of PD was also taken into account (see **Table 2** below for parameter estimates, and **Figure 1** for an illustration of the effects). Thus, it appears that the association between AS and SSs in unemployed adults is underlined by PD.

**Table 2:** Parameter estimates of the model examining the mediating role of psychological distress in the relationship between anticipated stigma and somatic symptoms.

Model	Estimate	*SE*	*p*	CI (lower)	CI (upper)
**Model without mediator**					
Intercept	30.14	5.35	<0.001	19.37	40.90
AS → SSs (*c*)	0.75	0.24	<0.01	0.26	1.23
*R*^2^ (*y,x*)	0.17				
**Model with mediator**					
Intercept	29.98	4.45	<0.001	21.03	38.93
*Model 1: PD as outcome variable*					
AS → PD (*a*)	0.70	0.13	<0.001	0.45	0.96
*Model 2: SS as outcome variable*					
PD → SSs (*b*)	1.07	0.23	<0.001	0.61	1.54
AS → SSs (*c’*)	-0.01	0.26	0.969	-0.53	0.51
Indirect effects (*a* x *b*)	0.76	0.20		0.42	1.23
*κ*^2^ for indirect effects	0.38	0.07		0.24	0.51
*R*^2^ (*m,x*)	0.40				
*R*^2^ (*y,m,x*)	0.44				

*We have also illustrated regression weights for *a, b, c*, and *c’* in **Figure 1**. For information, *R^2^ (y, x)* is the proportion of variance in y explained by *x, R^2^ (m, x)* is the proportion of variance in m explained by *x and m*. the 95% CI for *a × b* is obtained by the bias-corrected bootstrap with 5000 resamples. AS (anticipated stigma) is the predictor variable *(x)*, PD (psychological distress) is the mediator *(m)*, and SSs (somatic symptoms) is the outcome *(y)*. CI (lower), lower bound of 95% confidence interval; CI (upper), upper bound of 95% confidence interval*.

It can be seen that collectively, AS and PD account for 44% of the variance in SSs. Moreover, the Kappa squared effect size was found to be 0.38, which represents a large effect (Preacher and Kelley, 2011).

## Discussion

The current study aimed to investigate whether the anticipation of being stigmatized as an unemployed group member might drive the negative impact of unemployment on psychological and physical health. To address this question, we surveyed a sample of people experiencing unemployment to ascertain their self-reported levels of AS, PD, and SSs. As predicted, results showed that higher levels of AS were associated with both increased PD and increased SSs. Moreover, the effect of AS on SSs disappeared when PD was taken into account. This suggests that AS directly affects levels of PD, and that this then impacts on SSs, either in terms of actual differences in symptoms or in interpretation and report of symptoms. These findings add to our understanding of the relationship between unemployment and health, and suggest that interventions aiming to reduce AS, or improve coping mechanisms, would have the potential to offer health benefits to this cohort.

Our first finding, that anticipating greater levels of stigma predicted both increased PD and more SSs, fits with prior research on other stigmatized identities which shows they are associated with impaired well-being (e.g., Markowitz, 1998; Cluver et al., 2008; Hatzenbuehler et al., 2009; Quinn and Chaudoir, 2009; Earnshaw et al., 2013; Quinn et al., 2014). While it was known from previous research on unemployment that becoming unemployed leads to a new social identity that is stigmatized (e.g., Rantakeisu et al., 1997; Cullen and Hodgetts, 2001; Letkemann, 2002; Furåker and Blomsterberg, 2003; Lee et al., 2005), the specific link between unemployment stigma and impaired health has not been demonstrated before.

As such, our findings extend the stigmatized identities literature by demonstrating that this negative effect of AS on health also applies to the unemployed identity. Importantly, they also advance the unemployment literature by demonstrating the impact of a psychosocial factor – stigma – on the experience of impaired health in the unemployed group. This is particularly significant given that much research on stigma and unemployment has focused more on its impact on re-employment than its impact on health.

Moreover, and importantly, the current study also showed that there was an indirect effect of AS on SSs via PD. While there has been some evidence that unemployment is associated with poor physical health and that stigma is associated with poor health, we believe this is the first study showing that anticipated unemployment stigma affects PD and that this impacts self-reported physical health. Based on the existing literature, we suggest that stigma might exert such effects on both psychological and physical health by heightening levels of stress. While unemployment itself has been identified as a stressor (Linn et al., 1985; Wanberg, 2012), stigma is also a chronic stressor (Link and Phelan, 2006). Specifically, knowing that others are labeling and judging you can lead to withdrawal from support systems, which might otherwise provide a buffering effect, and hence increased stress (Markowitz, 1998). Of course, it must be noted that causality cannot be inferred when using mediation analysis on cross-sectional data. However, our predicted causal pathway is supported by previous research showing that more generally, stress can lead to PD (Schulz et al., 1995; Phillips et al., 2015) and that collectively, stress and PD can impact physical health (Cohen and Williamson, 1991; Hoge et al., 2007; Springer et al., 2007; Springer, 2009).

Finally, the current research also adds to the literature seeking to explain the association between unemployment and health problems, which demonstrates there is an impact of unemployment on health over and above any self-selection effect of people who are unwell into unemployment. In the current sample previous illness was uncommon, and moreover had no impact on the relationships between AS, PD, and SSs. Our findings add to a small but growing literature showing that, while self-selection may indeed exert an effect, this does not explain all health problems in the unemployed group. Rather, research now suggests there is also a distinct effect of unemployment on health (Korpi, 2001; Wanberg, 2012). The current study uniquely adds to our understanding in this area as it shows that the stigma related to being unemployed has an undesirable impact on one’s health, in addition to the documented impact that it already has on re-employment.

Although the study contributes in a novel way to multiple literatures, naturally it has some limitations that could be addressed in future research. First, the study provides cross-sectional data suggesting there is an indirect effect of AS on SSs through PD. Ideally, future research should investigate this longitudinally in order to establish causality. The sample in this study was also small, which is actually not untypical of research on the unemployed group, and difficulties with recruitment have been noted by other researchers (e.g., Blau et al., 2013). While our findings are very much in line with previous research on unemployment and health, nonetheless, it must be acknowledged that the small sample size means our study is underpowered. As such, in order to further advance research in this area, researchers must consider how best to promote recruitment of this vulnerable group, many of whom understandably do not wish to be asked about their experiences.

Future research should also incorporate objective measures of physical health outcomes, to disentangle whether PD is affecting actual SSs, the interpretation and reporting of same, or both. There has been some suggestion in the literature that effects may relate more so to perceptions of health than health itself (Cohen and Williamson, 1991), but it would be advisable to gather more evidence on this matter.

It would also be prudent to expand the measurement of stigma to include both experienced stigma and internalized stigma as well as AS. These measures have been identified as highly important in determining the impact of other concealable stigmatized identities (e.g., Earnshaw and Quinn, 2011), but have not yet been studied much in relation to the unemployed group. Internalized stigma in particular may be highly relevant to the unemployed group as unemployment is an identity that one acquires, having most likely had preconceptions about the group beforehand (Quinn and Earnshaw, 2011). Future research could also in some way take into account the economic climate, as previous findings suggest stigmatization may be highest when unemployment is low (Furåker and Blomsterberg, 2003; Ho et al., 2011; Karren and Sherman, 2012). Although unemployment is currently high in Ireland, the unemployment rate is also steadily dropping (CSO, 2015), and in any case, *perceptions* of this rate are perhaps more relevant to experienced, anticipated and internalized stigma, than the objective rate itself.

Finally, it would be ideal for future studies in this area to sample a more accurate gender representation relative to the entire unemployed group. In the present study, two thirds of our participants were female, which does not reflect the gender breakdown in the unemployment figures for Ireland (CSO, 2013). The most recent figures suggest that while women are less likely to be employed than men (55.9% of women are employed, compared to 65.7% of men), they are also less likely to be identified as *un*employed (9.9% of women compared to 13.9% of men). As such, the gender imbalance in the present study is likely to be due to women’s greater willingness to take part in surveys (Mackety, 2007). Although in the present study gender was not demonstrated to affect our results, more accurate gender representation in future research is important, as previous research has identified that unemployment can affect men and women differently, in terms of both stigma and PD outcomes. For example, unemployed men may feel more stigma than women due to work being more central to their sense of self (Kulik, 2000), and may experience more anxiety and mental health problems while women suffer more in terms of reduced self-esteem (Breslin and Breslin, 2013).

Overall, our findings are consistent with prior literature suggesting that stigma may be one missing piece of the puzzle in explaining health disparities between advantaged and disadvantaged groups (e.g., Eriksson et al., 2010; Quinn and Earnshaw, 2011), in this case, unemployment-related stigma. This empirical evidence is valuable as there has not been much research focusing on how the experience of stigmatization might help explain poor health in the context of unemployment. Unemployment is known to be stressful and to impact on psychological and physical health, and it is important to understand the processes by which it has these impacts.

In seeking to go beyond existing incomplete understandings of these processes, we argue it is vital to take into account social factors such as stigma. Stigma is especially important as it can be considered to exert effects at both the socio-structural level and the psychosocial level. That is, the low status of the unemployed group in the social structure leads people to experience actual discrimination due to unemployment-related stigma in the minds of others, but the expectation of such stigmatization and discrimination also has a negative effect on the well-being and functioning of the unemployed person at the psychological level. Aside from the clear theoretical implications, the research therefore has strong practical implications as well. For example, while stigmatization itself may be extremely hard to tackle, there is scope to expand job skills training to incorporate techniques for coping with stigmatization. In conclusion, our ability to boost people’s psychological and physical health during unemployment is likely to be far increased if we take into account social factors such as stigma.

## Conflict of Interest Statement

The authors declare that the research was conducted in the absence of any commercial or financial relationships that could be construed as a potential conflict of interest.
